# Transportation Barriers to Prenatal Care Among Black/African Americans: A Scoping Review

**DOI:** 10.1002/puh2.70128

**Published:** 2025-10-04

**Authors:** Ruth Appiah‐Kubi, Favorite Iradukunda, Madison Perry, Will Bazile, Paola Muwanga, Shannon C. Roberts

**Affiliations:** ^1^ Department of Health Promotion and Policy University of Massachusetts Amherst Amherst Massachusetts USA; ^2^ Elaine Marieb College of Nursing University of Massachusetts Amherst Amherst Massachusetts USA; ^3^ Department of Mechanical and Industrial Engineering University of Massachusetts Amherst Amherst Massachusetts USA; ^4^ Department of Chemical Engineering University of Massachusetts Amherst Amherst Massachusetts USA

**Keywords:** barriers to care, healthcare utilization, health service access, prenatal care, scoping review, transportation access

## Abstract

**Background:**

Reliable transportation is key to prenatal care (PNC) access and continuity. Disruptions in care due to transportation barriers may increase the risk of poor perinatal outcomes, especially among Black/African Americans.

**Objective:**

The objective of this review was to synthesize evidence on transportation barriers that Black/African Americans face and how this affects access and continued use of PNC.

**Methods:**

We conducted a literature search of articles published between 2012 and 2024 across six major databases, including PubMed, Web of Science, Scopus, EBSCO, PsycINFO, and CINAHL Complete, to identify relevant studies from April 24 to June 3, 2024.

**Results:**

Out of 240 records retrieved, 9 studies were included in this review. The findings are aligned with three major themes related to transportation barriers: initiation of PNC, continuity of PNC, and utilization of PNC. Seven studies indicated that the lack of access to reliable transportation contributed to delayed initiation of PNC, missed or cancelled appointments, and the need to reschedule appointments. Two studies found either a weak or no association between transportation and poor PNC attendance and utilization.

**Conclusions:**

Given the substantial gaps in the existing literature, further research is essential to fully understand the impact of transportation barriers on PNC among Black/African American populations.

## Graphical abstract

GRAPHICAL ABSTRACT

This scoping review maps existing evidence on transportation barriers to prenatal care among Black/African American populations in the United States. The PRISMA‐ScR flow diagram summarizes the identification, screening, and inclusion of studies relevant to this topic.

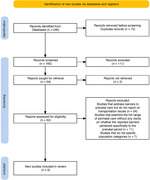

## 1 Background

Access to care during the prenatal period is critical for improving maternal and infant outcomes [1, 2]. Poor prenatal care (PNC) increases the risk of negative pregnancy outcomes, particularly for Black/African American mothers and infants, who experience higher mortality rates and pregnancy‐related complications compared to their White counterparts [2, 3, 4, 5, 6]. Infants born to non‐Hispanic Black/African Americans have a 2.5–2.8 times higher risk of mortality than infants born to non‐Hispanic Whites from complications, including those related to low birth weight, sudden infant death syndrome, unintentional injuries, and congenital malformations [7, 8, 9, 10]. Although overall infant mortality rates (IMRs) have declined between 1995 and 2020, significant racial disparities remain. Black non‐Hispanic infants experienced a higher mortality rate following preterm births compared to White non‐Hispanic infants, with an IMR of 31.09 (95% CI, 30.44–31.74) versus 21.81 (95% CI, 21.43–22.18) from 2018 to 2020 [11]. Pregnancy‐related complications are twice as prevalent among non‐Hispanic Black women compared to non‐Hispanic White women [12, 13, 14, 15, 16]. Moreover, Black/African American women are also at a greater risk of hypertension, preterm delivery, and antepartum hemorrhage, which could worsen pregnancy outcomes [17].

Although the exact causal relationship between PNC and poor maternal outcomes is not yet fully established, several studies have consistently shown a significant association between the use of PNC and maternal health outcomes [2, 6, 18, 19]. Studies indicate that women who receive inadequate PNC face an elevated risk of maternal and neonatal mortality, prematurity, low birth weight, and stillbirth [2, 17, 19, 20]. More recent data show a significant increase in the maternal mortality ratio among non‐Hispanic Black women who attended four or fewer prenatal visits [21]. In contrast, adequate PNC has been associated with lower rates of severe maternal morbidity and overall improvements in perinatal outcomes [2, 17, 20]. This is especially crucial for Black women, as access to timely PNC may mitigate pregnancy‐related mortality risks and enhance outcomes by enabling early detection and management of potential complications [2, 6, 18, 22, 23].

Despite the benefits associated with adequate PNC in improving maternal and infant outcomes, racial disparities in access to and use of PNC continue to persist [2, 6, 18]. Black/African Americans are less likely to complete the recommended PNC visits and may have insufficient and/or reduced first‐trimester PNC initiation than White women [2, 6, 23, 24]. Even for high‐risk pregnancies, Black women are two times less likely to receive adequate PNC (defined as care initiated by the fourth month of pregnancy and include at least 80% of the recommended number of visits), often due to factors such as a lack of insurance and potential biases within the healthcare system [6, 25, 26, 27]. In a study examining maternal factors contributing to late entry into PNC, the authors found that the percentage of women with delayed initiation of PNC was consistently greater among those with public insurance compared to those with private insurance. Among White non‐Hispanic and Asian women with private insurance, less than 1% began PNC late. On the other hand, more than 4% of White non‐Hispanic and Black women with public insurance experienced late entry into PNC [28]. In yet another study examining individual, household, and neighborhood characteristics influencing PNC utilization, the authors found that racial and ethnic minority populations, compared to White populations, had lower rates of first‐trimester PNC initiation and inadequate PNC. Socioeconomic status (SES), including education and income, explained 50.68%–79.92% of the PNC usage gap between Black and White populations and 37.50%–49.51% of the gap between Hispanic and White populations [29]. Provider bias, including perceptions of poor treatment based on race, refusal by providers to engage in meaningful conversations, and concerns about being dismissed or ignored, can negatively affect the use of PNC services [30, 31]. A strong and supportive relationship between patients and clinicians plays a key role in facilitating effective communication, influencing perceptions of care quality, and enhancing satisfaction with perinatal services [15, 32]. When interactions are strained and trust is lacking, patients may miss appointments or use PNC inconsistently, including delaying visits until emergencies arise [31]. Appointment availability and shorter wait times are also important for early PNC initiation and continuity of care [33].

Among these barriers, transportation is a key social determinant of health that plays a crucial role in PNC access and utilization [34]. More broadly, research on the impact of transportation on healthcare access and utilization has shown that transportation barriers can hinder access to care and result in delays in seeking medical attention [35]. For instance, in rural areas, non‐emergency medical care is often missed or delayed due to a lack of access to reliable transportation options [36]. Even in urban settings, low‐income individuals usually rely on public transportation but face challenges like inconsistent service, extended wait times, routes that fail to provide direct access to health facilities, requiring multiple transfers, and experiencing significant delays [37]. When public transportation is available, people living in cities and urban areas might not fully understand how the transportation system operates, which can limit their ability to access healthcare [38]. This challenge is even more pronounced for individuals with disabilities and those with chronic illnesses, for whom using public transportation can be physically demanding [35]. Missing healthcare appointments can result in worsening health conditions, higher rates of hospital readmissions, interruptions in ongoing care, and limited chances for diagnostic tests and early disease detection [39, 40].Additionally, lack of access to transportation can hinder one's ability to reach pharmacies or drugstores and consequently reduce medication adherence [41, 42].

Specifically, in maternal health, access to transportation may directly influence a pregnant woman's ability to access essential care, including PNC [34, 43]. Although prior studies have acknowledged transportation as a barrier to care, there remains a lack of synthesized evidence specifically examining how transportation challenges affect PNC access for Black/African American communities, who often face multiple systemic barriers. Moreover, although some studies suggest that transportation barriers may disproportionately impact these populations, the extent, nature, and implications of these challenges have not been systematically explored. To address this gap, this scoping review aimed to synthesize existing research on the availability, affordability, and reliability of transportation options and assess how these factors influence PNC access among Black/African Americans. Further, the review examined how transportation issues influence ongoing care decisions, including frequency, timeliness, and adherence to recommended prenatal visits. To our knowledge, this is the first review to synthesize evidence on transportation barriers and their effects on PNC in this population. In this review, we use the term Black/African Americans to refer to individuals of African descent, including African Americans and Black immigrants or persons with ancestral origins in any of the Black racial groups from Africa.

## 2 Methods

This scoping review was conducted to synthesize evidence on transportation barriers to PNC among Black/African Americans. Given the broad scope of our topic, we determined that a scoping review was the most appropriate method to map the available literature, identify research gaps, and provide a comprehensive overview of the evidence regarding transportation barriers faced by Black/African Americans [44, 45]. Our review followed the methodological guidelines recommended by the Joanna Briggs Institute for evidence synthesis [45]. We conducted the review following the Preferred Reporting Items for Systematic Reviews and Meta‐Analysis Protocols (PRISMA) for Scoping Reviews [46]. The review process involved (1) identifying the research question; (2) searching for relevant studies; (3) selecting studies using an iterative approach; (4) charting the data; and (5) collating, summarizing, and reporting the results.

### 2.1 Research Question

We utilized the PCC (population, concept, and context) framework to curate the review question and the title [45]. The population of interest includes Black/African American birthing individuals who use PNC. The concept focuses on assessing transportation barriers during PNC, within the context of healthcare and community settings. This framework led to the development of the research question: What evidence exists on transportation barriers faced by Black/African Americans during PNC, and how do these barriers impact their access to and use of PNC services?

### 2.2 Eligibility Criteria

In this review, we included studies that met the following criteria: (1) focused on or included populations identifying as Black/African Americans; (2) published in English between 2012 and 2024; (3) conducted in the United States to ensure contextual consistency in transportation systems, healthcare infrastructure, and racial dynamics. Because barriers to PNC and transportation challenges are shaped by country‐specific policies and social determinants, focusing solely on the U.S. allowed for a more accurate synthesis of factors influencing access to care among Black/African American populations within the U.S. healthcare context; (4) utilized qualitative, quantitative, or mixed‐methods designs; and (5) explored topics such as barriers and facilitators to PNC, racial disparities in PNC, geographic barriers to PNC, and the impact of transportation access on PNC utilization. We excluded the following: (1) studies that reported on social, structural, or geographic barriers to care, but results did not report on transportation; (2) studies that were commentaries, opinion articles, grey literature, or reviews; (3) studies that examined the full range of perinatal care without any clarity on whether the reported barriers pertained specifically to the prenatal period; and (4) studies that reported on barriers to PNC solely from the perspective of healthcare providers.

### 2.3 Information Sources

We identified studies for this scoping review by searching six major databases: PubMed, Web of Science, Scopus, EBSCO, APA PsycINFO, and CINAHL Complete. We also reviewed the reference lists of relevant articles to find additional suitable studies. The search was conducted between April 24, 2024, and June 3, 2024.

### 2.4 Search Strategy

Two researchers (S.C.R. and R.A.K.) initially developed the search terms, which were further refined using Medical Subject Headings (MeSH terms). We finalized the search strategy in consultation with a public health librarian using keywords and controlled variables to ensure comprehensive retrieval of relevant studies. Alerts were created in all the databases searched to inform researchers about new studies published on the topic. The search was conducted using the search string and Boolean operators: “barriers” AND “prenatal care” AND “Black” AND “women” AND “United States” AND “transportation.” Alternate terms were used for transportation (buses, subways, ride‐shares, cars, and bicycles) and Black/African Americans (Black women or African American women or women of color or Black females). These alternative terms were combined with the initial search terms to identify studies. To capture literature addressing experiential aspects of transportation and PNC, we included terms such as “perceptions,” “attitudes,” “views,” “feelings,” and “perspectives.” We selected these terms to reflect the broader concept of experiences, even though the word “experiences” itself was not included in the original search string. Search terms were iteratively refined, but keywords such as “transportation” and “prenatal care” were consistently maintained throughout the search. The identified studies were either imported directly into Mendeley or saved as RIS files and later imported into the reference manager. We filtered studies to exclude conference papers, notes, and reviews. The complete electronic search strategy for Web of Science is included in the Supporting Information Appendix.

### 2.5 Selection of Eligible Studies

The search yielded 240 records. Seventy‐five duplicates were identified and removed accordingly, leaving 165 records for initial screening. During this phase, titles and abstracts were evaluated independently and in duplicate by three researchers (R.A.K., M.P., and W.B.) based on predefined inclusion and exclusion criteria. This process narrowed the selection to 54 studies. Full texts of 52 records were reviewed concurrently and independently by the same researchers, consistent with the abstract and title screening process. Two records could not be retrieved. The screening and selection process was conducted in Rayyan. Any conflicts flagged by Rayyan were addressed and resolved through discussions among researchers. This was important for achieving consensus and ensuring a thorough and unbiased selection process [47]. After full‐text screening, 9 studies met the eligibility criteria and were included in this scoping review.

### 2.6 Data Extraction

Data extraction was performed concurrently and independently by two researchers (R.A.K. and P.M.). First, the data extraction form was developed by consensus among all researchers. This form was piloted during the initial literature review of this current scoping review. The form was refined throughout the extraction process to respond to preliminary findings and feedback and to ensure that all relevant information was included. The final extraction form (Table 1) captures the following headings: author(s)/publication year, study design, study population, sample size, study aim, and results.

**TABLE 1:** Summary of included papers.

Author(s) year of publication	Study design	Study population	Sample size (total) by specific race/ethnicity	Aim of study	Racial comparison conducted?	Results
Alhalel et al. [48]	Qualitative study (interview)	Clinical care team members who work with pregnant patients and Black or African American who were currently pregnant or had recently given birth	31 **Clinical care team** White (2) Hispanic or Latino (2) Black or African American (11) Asian/Pacific Islander (3) Other (2) Unanswered (1) **Patients** Black or African American (8) Native American or American Indian (1) Unanswered (1)	Explore patient experiences of perinatal care and clinicians’ perspectives on what they consider barriers to Black maternal health disparities	No	Transportation was identified as a major barrier to accessing services. Participants reported that unreliable or inaccessible transit options contributed to stress, disrupted scheduling, delayed appointments, and limited engagement with care. Consistent and supportive transportation services were seen as essential for improving access and reducing anxiety
Fryer et al. [54]	Secondary analysis of a prospective cohort study (phone and home interviews and chart reviews)	Women who had a live birth of an infant greater than 20 weeks gestation, had a subsequent pregnancy during the 2‐year follow‐up period, and answered one question regarding barriers to prenatal care in subsequent pregnancy	298 Black (151) White (72) Latinx (75)	Describe barriers to prenatal care	Yes‐both descriptive and statistical comparison	Transportation was consistently identified as a barrier to prenatal care. Notably, 22 of the 30 participants who reported transportation‐related challenges were Black, with several indicating they had no reliable means of reaching clinics for scheduled appointments. Additionally, Black women faced nearly twice as many obstacles to obtaining prenatal care compared to White women, including transportation barriers that limited their ability to attend appointments with an adjusted rate ratio of 1.89 [1.2, 3.0]
Holcomb et al. [53]	Retrospective cohort study	Pregnant women delivering at ≥24 weeks’ gestation in a large inner‐city public hospital system	**Cohort 1‐established prenatal care during current pregnancy** Hispanic (7299) Non‐Hispanic Black (1528) Non‐Hispanic White (407) Other (254) **Cohort 2 presenting for delivery without established PNC** Hispanic (210) Non‐Hispanic Black (59) Non‐Hispanic White (43) Other (14)	Assess geographic barriers to prenatal care	No	Women without prenatal care coverage were more likely to depend on public transportation (e.g., buses) to reach prenatal clinics. Their trips took significantly longer and involved more stops compared to those with coverage (42 min vs. 30‐min travel time; 29 vs. 17 stops, respectively)
Krukowski et al. [56]	Secondary analysis of PRAMS survey	Women who responded to the PRAMS survey in 2016	31,642 White (18,616) Black (5565) Asian (1941) Native North American (1219) Other races (1685) Mixed race (1310) Hispanic (6193)	Identify predictors of early prenatal care, including perceptions of early prenatal care initiation, and describe perceived barriers to receiving early prenatal care	Yes‐statistical comparison	Transportation barriers were associated with reduced access to early prenatal care. Black women had significantly higher odds (OR: 2.9; 95% CI: 1.7–5.2) of having no access to transportation that affected their access to early prenatal care
Lee [55]	Cross‐sectional (secondary analysis of Pregnancy Risk Assessment Monitoring Systems Survey (PRAMS)	All individuals with live birth and who reported any experiences of cancellation or delay in prenatal care	11,427 White (4791) Black (2038) Asian (999) Other (934) Hispanic (2589)	Examine various factors that are associated with disparities and interruptions to prenatal care	Yes‐statistical comparison	Among Black individuals, unreliable transportation was strongly linked to more frequent delays or cancellations of prenatal appointments, as indicated by an adjusted prevalence ratio of 2.26 (95% CI: 1.42–3.58; *p* = 0.001) compared to White individuals
Lynch et al. [51]	Randomized controlled trial	Pregnant Medicaid recipients <32 weeks gestation	143 Intervention group White non‐Hispanic (9) Black non‐Hispanic (54) Hispanic (3) Other non‐Hispanic race (5) Control group White non‐Hispanic (12) Black non‐Hispanic (56) Hispanic (3) Other non‐Hispanic race (1)	Assess the influence of modernized nonemergency medical transportation services prenatal care utilization	No	Participants in the intervention group reported higher satisfaction with transportation services (83.8% vs. 68.8%), showing a 14.8% improvement. However, this increase in satisfaction did not translate into a meaningful difference in the adequacy of prenatal care utilization (APNCU), with a risk difference of 2.1% (95% CI: −14.0 to 18.2). Thus, access to modernized nonemergency transportation services had no significant effect on prenatal care utilization
Mazul et al. [49]	Focus group	African American women who had attended at least one prenatal visit in their current pregnancy	31 African American (31)	Discuss perspectives on barriers and facilitators to prenatal care	Not applicable‐study focused exclusively on African Americans	Participants reported significant challenges in accessing prenatal care due to transportation issues. Many lacked personal vehicles and depended on friends, public transit, or health plan‐provided rides. These options often proved unreliable or inconvenient, especially during extreme weather or late pregnancy. Delays with transportation services frequently led to missed or rescheduled appointments, adding stress, and limiting care access
Reid et al. [50]	Mixed methods (survey and semi‐structured interviews	Postpartum women who received initial prenatal care at 14 weeks or later or no prenatal care and birthed at hospitals that provide care for high‐risk pregnancies	55 Hispanic (28) Non‐Hispanic White (12) Non‐Hispanic Black (9) Asian (1) Other (5)	Explore participant's views on quality of care and access during the prenatal	No	Transportation barriers, such as lack of access to a personal vehicle or public transportation and long travel times to clinics, were identified as challenges to accessing prenatal care. These issues sometimes resulted in delays in care, including postponed tests
Wolf et al. [52]	Retrospective case–control study	Mothers delivering at an academic safety health system between May 2017 and May 2018 and children 1–3 years old by 2019 who attended at least one well‐child visits between 2 and 6 months born at this academic safety‐net‐health system	891 mothers **Mother** Cases ≤50% visit adherence or initiation after 5 months White (54) Black (114) Hispanic (107) Other (22) Controls ≥80% adherence or initiation White (172) Black (157) Hispanic (152) Asian (7) Other (106)	Assess factors contributing to poor prenatal care	No	Transportation difficulty was not significantly associated with poor prenatal care attendance

### 2.7 Synthesis Method

Data from this scoping review were synthesized using a convergent integrated approach [44]. This method is appropriate for synthesizing data from reviews that include both quantitative and qualitative data types. First, the quantitative data were transformed into qualitative data by extracting and converting it into textual descriptions and then organizing the descriptions into thematic categories. Following this, data from both qualitative and quantitative studies were integrated to inform the overall findings [44]. The development of the initial codebook to capture emerging themes occurred concurrently with the data transformation process. Two researchers (R.A.K. and P.M.) independently reviewed all included studies to develop codes and identify patterns and emerging themes related to the overall study aim and research question. Thematic findings were synthesized and grouped into three overarching categories, each reflecting aspects of PNC impacted by transportation‐related barriers: (1) initiation of prenatal care; (2) continuity of prenatal care; and (3) utilization of prenatal care.

## 3 Results

### 3.1 Search Outcome

**FIGURE 1:**
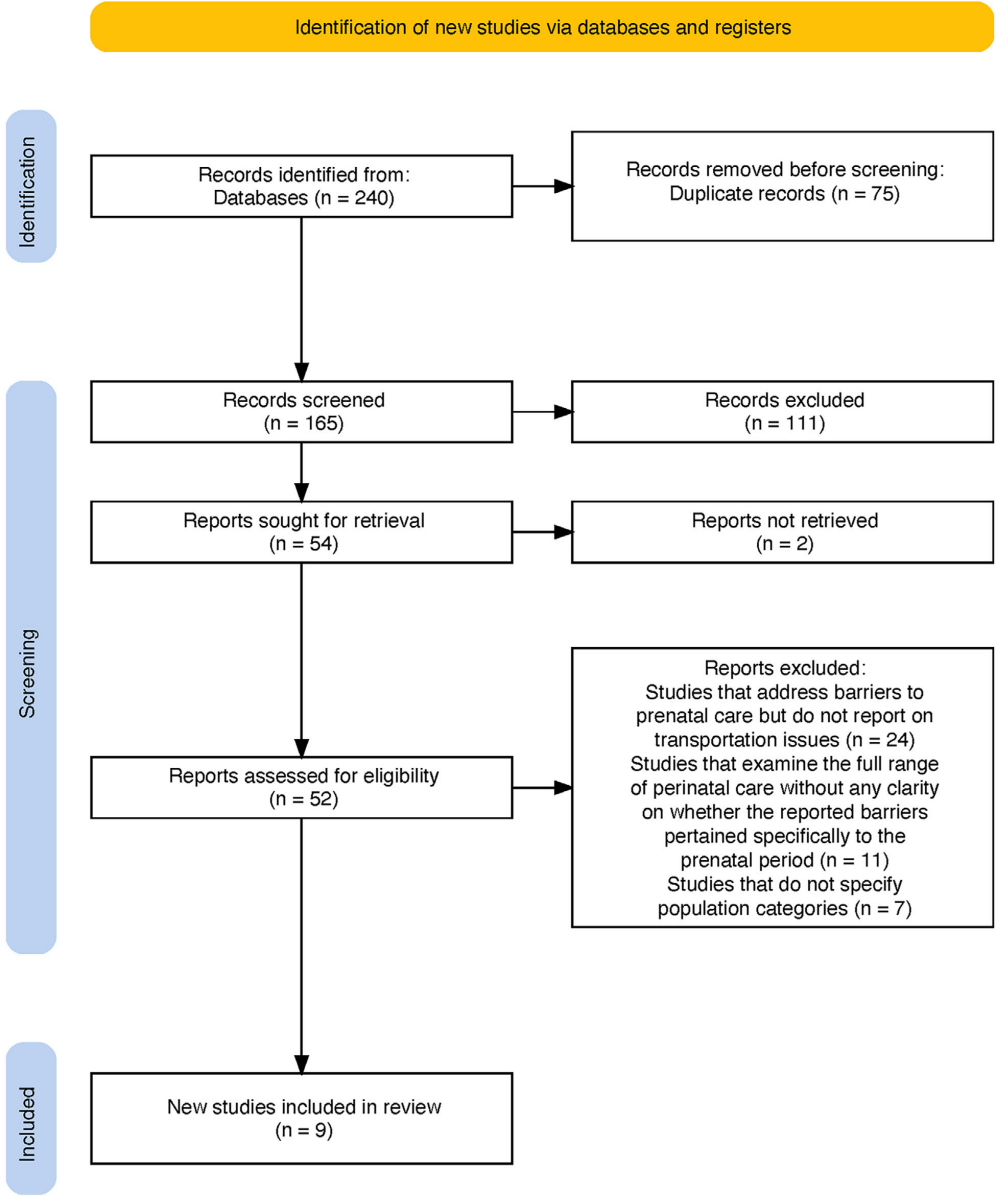
PRISMA‐ScR flow diagram for study selection.

This scoping review involved searching six major databases to identify relevant studies. We identified 240 records. A total of 52 records underwent full‐text screening. Common reasons for exclusion were study design (e.g., systematic reviews) and studies that report on barriers to PNC without focusing on transportation. The process and outcomes of study selection are illustrated in the PRISMA‐ScR flow diagram (Figure 1).

### 3.2 Characteristics of Studies

The included studies comprise various study designs, population demographics, and outcomes of interest. Table 1 provides a summary of key data and findings from these studies.

### 3.3 Study Types and Settings

The included studies employed a diverse range of research designs across various settings to highlight the influence of transportation on PNC for Black/African Americans. Two studies used qualitative design methods, drawing on structured interviews and focus groups to gather data among African American/Black individuals and low‐income African American women, respectively [48, 49]. Reid et al. [50] utilized a mixed‐methods approach with surveys and semi‐structured interviews among postpartum women who established PNC beyond 14 weeks gestation. Lynch et al. [51] utilized a randomized controlled trial study design among Medicaid recipients in Franklin County, Ohio. Wolf et al. [52] conducted a retrospective case–control analysis using data from patients within the Virginia Commonwealth Health System. A total of two studies were designed as cohort studies [53, 54]. Fryer et al. [54] conducted a secondary analysis of the Community Child Health Research Network Study, a multisite prospective cohort study among women who had subsequent pregnancies. Holcomb et al. [53] conducted a retrospective cohort study among pregnant women who delivered at 24 weeks gestation or later in a large public hospital system. Two authors performed secondary analysis of larger datasets [55, 56]. Krukowski et al. [56] conducted a secondary analysis of the 2016 Pregnancy Risk Assessment Monitoring Systems (PRAMS) survey data, including women from across the U.S. Lee [55] performed a cross‐sectional analysis using 2020–2021 data from the PRAMS. Studies included in our review were conducted in diverse settings, including hospitals specializing in high‐risk pregnancies, inner‐city public hospital systems, local communities across states, national level, academic safety‐net health systems.

One study examined the impact of transportation as the primary variable of interest on PNC utilization [51]. Six studies analyzed secondary data, mainly from surveys examining maternal health issues, such as past pregnancy experiences and health‐seeking behaviors, particularly among recent mothers [52, 53, 54, 55, 56]. Observational studies, including case–control, cross‐sectional, and cohort designs, were employed to identify risk factors associated with poor PNC attendance. Another study explored the experiences/perceptions of PNC barriers from the perspective of both birthing individuals and perinatal care team members who care for this population [48].

### 3.4 Population

The populations studied were diverse. Participants included pregnant individuals and those in the postpartum period [49, 50, 52, 53, 55, 56]. One study included clinical care team members [48]. Participants varied by insurance type, with some studies specifically focusing on pregnant Medicaid recipients [51]. Of the 9 studies in this review, two specifically focused on Black/African Americans [48, 49], whereas three emphasized racial and ethnic minorities more broadly [49, 54, 55]. The remaining studies addressed broader populations with diverse racial and ethnic backgrounds [50, 51, 52, 53, 56].

### 3.5 Outcomes and Key Measures

Although transportation was not always the primary variable of interest, several studies in this review captured its influence through both qualitative and quantitative means. Lynch et al. [51], the only randomized clinical trial, assessed the impact of modern‐nonemergency transportation services on PNC utilization using the Adequacy of Prenatal Care Utilization (APNCU) Index. Others focused on exploring barriers to care. For example, Alhalel et al. [48] and Mazul et al. [49] explored perceptions and experiences related to PNC barriers. Reid et al. [50] identified early PNC barriers among postpartum women. Three studies utilized electronic health records and survey data to examine the adequacy of PNC across different care‐seeking patterns [52, 53, 54]. Lee [55] and Krukowski et al. [56] utilized PRAMS survey data to identify delays or missed appointments related to transportation and to investigate predictors of early PNC.

### 3.6 Synthesis of Results Into Themes

Three major themes described the transportation barriers and their impact on PNC access and use: initiation of prenatal care, continuity of prenatal care, and utilization of prenatal care.

### 3.7 Initiation of PNC

This theme describes the initiation of PNC, defined as the timing of a pregnant individual's first clinical assessment during pregnancy according to the Kotelchuck's APNCU Index [27]. Although evidence on optimal timing is limited, current clinical guidelines recommends offering the initial visit before 10 weeks of gestation or shortly after pregnancy is confirmed [57]. Interviews from Alhalel et al. [48] revealed that transportation issues contributed to delays in starting PNC. Healthcare providers serving Black/African American individuals observed a trend of missed appointments among this population, which they attributed to the lack of access to personal vehicles. They also noted that this group had fewer appointments and were more likely to delay initiation of care. Krukowski et al. [56] analyzed the 2016 PRAMS dataset and found that Black/African American individuals, who comprised 13.5% of the study population, were 2.9 times more likely (95% CI: 1.7–5.2) than their counterparts to miss early PNC due to transportation issues. Besides, Holcomb et al. [53] found that study participants who resided in communities with a heavy reliance on public transportation had not established PNC. Likewise, those with longer travel times to the nearest prenatal clinic (42 min compared to 30 min; *p* = 0.005) and more bus stops along the route (29 vs. 17; *p* < 0.001) made up a substantial portion of those who did not receive PNC. African American women were disproportionately represented among those lacking PNC. The study by Fryer et al. [54] further revealed that transportation was a common barrier among Black women accessing PNC. Several study participants could not attend prenatal appointments due to a lack of transportation. Transportation‐related difficulties were a frequent concern in this study, particularly among Black women, who made up 22 of the 30 participants reporting such barriers with an adjusted rate ratio of 1.89 [1.2, 3.0].

These findings highlight how transportation challenges, such as limited access to personal vehicles, can delay the initiation of PNC and point to a potentially disproportionate burden of such barriers among Black/African American individuals. However, such barriers do not end at the first visit. In the next theme, we examine how transportation constraints continue to affect ongoing PNC attendance throughout pregnancy.

### 3.8 Continuity of PNC

Initiating PNC does not always guarantee continued use. Even when prenatal care is geographically or structurally accessible, challenges related to available transportation options, such as reliability, scheduling, or cost, may still hinder individuals from frequently attending their appointments. In this theme, we explore how transportation‐related barriers affect the actual use of PNC once care is established. We define continuity of PNC as the ability of pregnant individuals to present for scheduled prenatal visits consistently following the initiation of care.

Transportation barriers, including limited options and difficulties with available modes, significantly affected attendance to PNC. In Mazul et al.’s [49] study, participants who did not own a personal vehicle reported challenges stemming from relying on friends, family, public buses, and HMO‐provided cabs for travel to prenatal appointments. Each of these modes of transportation presented a set of challenges, including constraints on provider choice, exposure to extreme weather, and unpredictable or poorly coordinated pickup and drop‐off times that negatively impacted appointment attendance. Participants who relied on friends and family often had to choose providers based on proximity to their support network. HMO‐provided cabs frequently arrived either too early or too late. When cabs arrived late, it often led to delays and the need to reschedule appointments. In line with these findings, Alhalel et al. [48] observed that transportation barriers were more likely to hinder attendance to prenatal appointments among Black/African American individuals. Participants in this study reported frequently postponing appointments and experiencing substantial delays, often due to the extensive travel time related to public transportation [48].

Analysis of larger datasets has frequently identified unreliable transportation as a significant predictor of repeated cancellations and delays in PNC appointments. In Lee's [55] analysis of PRAMS survey data from 2020 through 2021, absence of reliable transportation was a significant factor leading to frequent cancellations of prenatal appointments. Black individuals reported higher rates of cancellations or delays due to unreliable transportation options, with an adjusted prevalence ratio of 2.26 (95% CI: 1.42–3.58; *p* < 0.001). Reid et al. [50] characterized transportation barriers as community/social conditions affecting access to PNC. Several participants in this study reported difficulties reaching health facilities for PNC due to limited access to reliable transportation. Many more reported that their localities lacked essential transit options, such as buses or taxis, making it difficult to continually access PNC. Furthermore, some could not use ride‐sharing services due to eligibility or payment requirements such as having a bank account. A notable proportion of participants cited a lack of transportation as the reason they could not visit clinics or doctors’ offices (27%). Others reported that the clinic was too far (20%) or that travel time from home exceeded 30 min (33%). Transportation barriers, especially at the community level, such as the absence of public transit routes, limted availablity of buses or taxis, long distances to clinics, and long travel times, can affect follow‐up care. Addressing community‐level transportation gaps, including investing in neighborhood‐based transit solutions and supportive transportation programs, can improve access to PNC with lasting impact on maternal outcomes.

Although these studies highlighted the effects of transportation barriers on frequent attendance to PNC, Wolf et al. [52] in contrast reported no significant association between transportation‐related barriers and poor attendance to PNC in their retrospective case–control study. The study assessed a range of risk factors that influence PNC attendance. After adjusting for other variables, transportation barriers did not remain statistically significant. This suggests that transportation barriers had only a limited impact on PNC attendance.

These differences across studies highlight the importance of considering broader contextual and structural factors when examining PNC attendance.

### 3.9 Utilization of PNC

The previous theme emphasized continuity, specifically sustained engagement with prenatal services. In contrast, the theme of PNC utilization shifts attention to whether that engagement meets recommended standards. In this theme, we refer to utilization of PNC as the extent to which a pregnant individual attends the recommended number of prenatal visits during pregnancy. Based on the APNCU Index, this dimension evaluates visit frequency relative to gestational age at delivery. It classifies care as inadequate, intermediate, adequate, or adequate plus [27].

Lynch et al. [51] evaluated the effect of an enhanced smart transportation (EST) intervention and found no significant impact on PNC utilization. In this randomized pilot trial, the intervention group was compared to a standard non‐emergency medical transportation group (usual transportation). Both groups showed similar levels of prenatal care utilization, rated as adequate and adequate plus with 68.8% of the EST group and 66.7% of the standard group meeting these criteria. Overall, 76.1% of the study participants identified as Black/African American. Findings from this intervention study suggest that improving transportation access, while necessary, may not be sufficient to increase PNC utilization. Comparable rates of adequate care across groups may point to the influence of additional structural and individual‐level factors that shape consistent engagement with PNC for Black/African American populations.

## 4 Discussion

This scoping review sought to synthesize literature regarding the influence of transportation on PNC among Black/African Americans. Three major themes emerged: initiation of PNC, continuity of PNC, and utilization of PNC which together define the multifaceted impact of transportation barriers on maternal health among Black/African American women.

Across the studies reviewed, transportation barriers, such as lack of personal vehicles, limited public transit, long travel distances, and associated costs, were commonly cited as factors that hindered timely access to care, contributed to missed or delayed appointments, and compromised the ability to receive consistent and adequate PNC. Consistent with existing literature, limited transportation options reduce access to healthcare services, especially in underserved communities. For example, Syed et al. [37] found that transportation is a critical barrier to healthcare access across low‐income populations, a finding highlighted in Reid et al. [50] and Fryer et al. [54], who observed that Black women frequently encounter systemic limitations in public transit or ride‐sharing services. Another study conducted in rural Kansas reported that women who traveled ≥20 mi for care had significantly fewer visits and services than those traveling shorter distances [58]. This finding is reinforced by national data from 2017, which estimated that approximately 5.8 million Americans representing 1.8% of the population delayed medical care due to a lack of transportation, with low‐income and minority populations bearing the greatest burden [59].

These disruptions are particularly concerning given the time‐sensitive nature of PNC, where consistent monitoring and timely medical attention are essential for optimal maternal and fetal outcomes [22, 23]. Previous research highlights similar concerns. One systematic review found that transportation barriers substantially hinder access to care and recommended strategic solutions such as siting clinics near transit hubs and extending clinic hours [60]. Likewise, other studies emphasize that interventions aimed at improving local transportation infrastructure or relocating services closer to communities can enhance early PNC uptake among Black populations and help reduce racial disparities in first‐trimester care initiation [61].

Although the negative impact of transportation on care adequacy may appear limited when assessed in isolation, its significance becomes clearer when examined alongside other compounding barriers [62]. In one study, travel time alone was found to be a moderate predictor of missed visits after accounting for other factors. A recent qualitative study also found that transportation difficulties were one of several interlocking barriers that made even attending any prenatal visit impossible for disadvantaged women [63]. Transportation burdens often intersect with other barriers (e.g., inflexible work schedules and childcare) and amplify their effect [64, 65]. Our synthesis contributes important insights by highlighting community‐level infrastructure and social factors, such as not having a bank account, that restrict the ability to access rides and reach care [50].

Many studies in this review found transportation to significantly influence prenatal care;however, a few did not report such association. Lynch et al. [51] observed that even with enhanced transportation support, improvements in utilization were limited. Previous systematic reviews on non‐emergency medical transportation services have also found that more rigorous studies revealed low patient utilization of transportation services and inconsistent effects on health outcomes and service use [66]. A clinical trial evaluating ride‐sharing services for primary care corroborates these findings, as adoption remained low and no meaningful reduction in missed appointments was observed [67]. These results also support critiques of traditional adequacy indices, such as the APNCU Index, which may fail to account for the systemic inequities that shape PNC patterns [68]. Hence, although providing transportation options may help, truly improving adequate PNC likely requires broader systemic changes to reach especially isolated and low‐resource communities [60].

Transportation‐related delays, whether from unreliable transit, inaccessible ride‐hailing platforms, or lack of nearby services, disrupt care continuity and heighten the risk of maternal complications. In one study, very long travel times to the nearest delivery facility (≥120 min) were associated with a doubling of stillbirth and preterm birth risks. The authors also found that these adverse outcomes were strongest among women with inadequate antenatal care, suggesting that lengthy travel compounds the harm of missed visits [69].

Our findings support more recent scholarship calling for better equity‐focused approaches to maternal health frameworks that shift away from individual‐level explanations, such as noncompliance, toward upstream structural factors, including neighborhood disinvestment and systemic racism [70].

### 4.1 Limitations

This scoping review should be considered with certain limitations in mind, including those related to both the review process itself and studies included. Although this review provides some evidence on transportation barriers and their impact on PNC access and continued use among Black/African Americans, it was sometimes unclear how many Black/African Americans reported inadequate or a lack of access to PNC due to transportation constraints. This lack of clarity may stem from the inclusion of diverse populations in the study samples, which makes it difficult to isolate the experiences of Black/African Americans. Only two studies focused exclusively on Black/African American populations. In several others with broader demographic representation, individuals from this group comprised a limited proportion of participants. As a result, the extent to which the findings reflect the specific transportation barriers faced by Black/African Americans when seeking and using PNC may be limited. The included studies also assessed multiple factors that affect PNC. Only one study specifically focused on transportation as a primary variable. This may limit our understanding of the specific role that transportation plays in PNC access and use.

Another key limitation of this review is the heterogeneity of the study populations, which may contribute to variations in findings. The included studies focus on populations from different geographic regions, such as rural Appalachia and urban Columbus, Ohio, with distinct socioeconomic conditions and levels of healthcare access. These contextual differences likely shape transportation barriers and PNC experiences in distinct ways. Although this review highlights common themes, the challenges faced by different subgroups may not be uniform.

Studies that utilized surveys may have relied on self‐reported data, which may be prone to reporting biases such as recall bias and providing socially desirable responses. Similarly, survey questions that restricted responses to binary options, such as yes or no, may not capture the full complexity of transportation barriers affecting PNC and may lack the context needed to fully understand the influence of transportation barriers on access and use. Several studies suggested that transportation barriers may negatively influence access to and use of PNC; however, the specific mechanisms driving these associations and their impact on actual use of PNC were sometimes unclear. Finally, most studies did not specify how transportation difficulties were measured, or which modes were assessed, limiting the ability to compare findings across studies.

## 5 Conclusions

One of the key contributions of this review is the identification of persistent gaps in the literature surrounding transportation barriers to PNC among Black/African American populations. This gap is a critical finding, as it reflects longstanding areas of under‐exploration in maternal health research and highlights the urgent need for equity‐focused inquiry and policy development. The findings also suggest that unreliable transportation may lead to limited access to PNC. Thus, pregnant individuals may experience inadequate care, missed appointments, cancellations, and rescheduling. Addressing these transportation barriers is critical to ensuring timely and consistent access to PNC and improved health outcomes for both mother and baby. Transportation‐related barriers to PNC require a coordinated, multisectoral response from the entire perinatal care team. These findings emphasize the importance of integrating transportation considerations into maternal health planning and service delivery, particularly for historically marginalized populations. Perinatal care providers, including nurses, midwives, obstetricians, social workers, and case managers, can play a critical role in identifying transportation challenges and tailoring care coordination accordingly. Although the evidence base for non‐emergency medical transportation remains limited, particularly in terms of demonstrating effectiveness in increasing PNC utilization, emerging strategies offer promising avenues. These include providing transportation vouchers or subsidies and aligning appointment scheduling with available transit options. Moreover, home visiting programs, mobile clinics, and telehealth initiatives may reduce travel‐related barriers for pregnant individuals, especially in rural or underserved areas. Policymakers and healthcare administrators should prioritize investment in community‐based models of PNC that incorporate transportation support. Collaborations across health, transportation, and social service sectors can help design systems that are responsive to the mobility needs of pregnant people. Continued evaluation and implementation research are necessary to identify which transportation solutions are most feasible, cost‐effective, and impactful in improving PNC access and outcomes.

### 5.1 Recommendations for Future Research

Despite growing attention to transportation as a barrier to PNC, few studies have examined its effects with sufficient depth, specificity, or cultural relevance among Black/African American populations. To advance understanding and inform equitable interventions, future research should more deeply investigate how transportation influences PNC experiences and outcomes within these communities. Studies should aim to disaggregate data by race and ethnicity, explore structural and community‐level transportation challenges, and incorporate the lived experiences of Black/African American individuals. Such efforts are vital to informing targeted interventions and advancing equity in maternal health outcomes. Moreover, studies should explore how these challenges affect PNC initiation, the consistency of attendance, and the overall adequacy of care. Research should also prioritize the development and evaluation of tailored interventions designed to mitigate these barriers. Recognizing the limited evidence on intervention effectiveness identified in this review, implementation research, including mixed‐methods and community‐based participatory approaches, will be essential to generating actionable insights and informing policy and practice.

## Author Contributions

*Conceptualization*: Favorite Iradukunda, Shannon C. Roberts, Ruth Appiah‐Kubi. *Methodology*: Shannon C. Roberts, Ruth Appiah‐Kubi. *Search Strategy Development*: Shannon C. Roberts, Ruth Appiah‐Kubi. *Literature Search and Screening*: Ruth Appiah‐Kubi, Madison Perry, Will Bazile. *Data Extraction and Charting*: Ruth Appiah‐Kubi, Paola Muwanga. *Formal Analysis and Synthesis*: Ruth Appiah‐Kubi. *Writing Original Draft Preparation*: Ruth Appiah‐Kubi. *Writing Review & Editing*: All authors. *Supervision*: Shannon C. Roberts. *Project Administration*: Shannon C. Roberts. *Validation*: All authors. *Visualization*: Ruth Appiah‐Kubi. *Resources*: Shannon C. Roberts.

## Conflicts of Interest

The authors declare no conflicts of interest.

## Supporting information




**Supporting File 1:** Appendix 1– Full Electronic Search for Web of Science.

## Data Availability

Data sharing is not applicable to this article as no new data were created or analyzed in this study.
