# Supplementary material for: Transportation Barriers to Prenatal Care Among Black/African Americans: A Scoping Review
Source: Public Health Chall. 2025 Oct 4;4(4):e70128. doi: 10.1002/puh2.70128 (PMC12495888; doi:10.1002/puh2.70128)
Supplement: Supplementary file 1 — Supporting File 1: Appendix 1– Full Electronic Search for Web of Science. [file PUH2-4-e70128-s001.docx]

**Appendix 1- Full Electronic Search for Web of Science**

The search query was structured as follows: "barriers" AND "Prenatal Care" AND "Black" AND "Women" AND "United States" AND “transportation”. We applied filters to include only articles published between 2012 and 2024 and those published in the United States. This resulted in 5 records. We used the analyzing feature to review and categorize results using MeSH headings. Relevant analysis blocks were reviewed and further refined. The Mesh headings selected include prenatal, pregnancy, health services, health service access, transportation, healthcare utilization, health service accessibility, maternal care, patient transport, demography, African American, and prenatal care. This process resulted in all 5 records being retained. The data was then saved and exported as an RIS file for use with the Mendeley reference manager. Alerts were also set up to track new publications and updates related to the search query.
